# Activated inhibition in regulating excitability

**DOI:** 10.1113/JP274196

**Published:** 2017-04-11

**Authors:** Joern R. Steinert

**Affiliations:** ^1^ MRC Toxicology Unit, Hodgkin BuildingUniversity of LeicesterLancaster RoadLeicesterLE1 9HNUK

**Keywords:** metabotropic glutamate receptor, neuronal excitability, presynaptic inhibition

The development of the nervous system requires precise and coordinated activities of multiple signalling pathways. In order for a neurone to become a functional component of the network it needs to know its abilities and limitations. These two main features of any neurone are determined by the set point of the excitability, which regulates its operational range. This in turn is determined by sodium, calcium (Ca^2+^) and potassium (K^+^) channels, the activities of which depend on the membrane voltage, which in turn is affected by synaptic inputs.

The auditory system consists of a network of nuclei that require highly tuned firing activities of neurones in order to detect and process sound information. It is a challenging process to compute the azimuth location of a sound source by integrating the acoustic stimuli from both ears. This involves the lower brain regions that contain auditory nuclei such as the cochlea, cochlear nucleus and superior olivary nucleus (which itself receives information via the medial nucleus of the trapezoid body) before eventually the auditory cortex is reached. The rat auditory pathway has to undergo several plasticity steps to continuously adapt to changes in environment, but it also experiences a great amount of developmental plasticity, in particular during the time period of hearing onset at around the end of postnatal week 2. A fundamental goal for developmental neuroscience is to understand how neurones assemble into the network to carry out their specific functions. Some progress has been made in understanding the general neuronal functions, but much less is known about other central features of developing neural circuits.

The study by Ye *et al*. (2017) in this issue of *The Journal of Physiology* precisely addresses the question: ‘What pathways could contribute to the development and changes in excitability of the sensory inner hair cells (IHCs) within the cochlea?’ These cells are important and essential relays to auditory neurones that transmit sound information to the central nervous system. The authors employed electrophysiological methods and pharmacology to characterise synaptic activities of IHC before maturation, i.e. hearing onset. IHCs release the excitatory neurotransmitter glutamate, which activates ionotropic glutamate receptors (iGluRs) on the afferent dendrites of neurones within the spiral ganglion. Importantly, this released glutamate can also activate G‐protein‐coupled metabotropic glutamate receptors (mGluRs), in general expressed either post‐ or presynaptically. The activation of presynaptic mGluRs has been shown to act as a feedback loop and is involved in neuronal plasticity and development (Maiese *et al*. 2005; Kullmann & Lamsa, 2008). The study by Ye *et al*. characterised the presynaptic involvement of group I mGluR1, the activation of which increases the strength of inhibition by enhancing release of the inhibitory neurotransmitter acetylcholine (ACh) of efferent terminals that contact IHCs. This study uses pharmacology to enhance mGluR activity and shows increases in efferent inhibition by potentiating ACh release. The authors went further to show that endogenous release of glutamate also involves the same pathway in a process called glutamate spill‐over, which can activate not only synaptic but also extrasynaptic receptors. The spill‐over, which is usually limited by glutamate uptake systems, is enhanced following high frequency firing activity (up to 10 Hz) such as seen in immature IHCs (Sendin *et al*. 2014) thereby providing a plausible basis for physiological activity‐dependent signalling of this kind.

But what are the underlying mechanisms for such signalling? The mGluR‐mediated enhanced inhibition could arise from activation of Ca^2+^ channels that enhance transmitter release (such as P/Q and N‐type Ca^2+^ channels) or inhibition of K^+^ currents that repolarise the membrane potential (such as large conductance Ca^2+^‐activated BK channels). This Ca^2+^ and K^+^ channel‐dependent modulation of release is present at the developing cochlea and might be responsible for the observations made by Ye *et al*. Other molecular pathways that have not been addressed in this study involve the modulatory activities of nitric oxide (Steinert *et al*. 2008; Kong *et al*. 2013) or endocannabinoids (Chi & Kandler, 2012), both reported in the auditory system. Cannabinoid receptors (CB_1_) are present presynaptically in the cochlea nucleus and their activation has been shown to inhibit Ca^2+^ influx through L‐type Ca^2+^ channels and thereby reduce release. Moreover, given the high firing activities of IHCs before reaching maturity and their high‐Ca^2+^‐triggered nNOS activity (Shen *et al*. 2005), the subsequently enhanced production of nitric oxide and nitrergic regulation of K^+^ channels could potentially contribute to a developmental adjustment of excitability.

Regardless of the mechanisms, the study by Ye *et al*. strongly supports the notion that activation of mGluRs present presynaptically at efferent terminals at the IHC synapse contributes to a physiological signalling during the development of this neuronal connection before hearing onset and synapse maturation. Thus, the high spike rates reported for the IHCs allow for a glutamate spill‐over and activated mGluR signalling, which represents an essential and new signalling pathway in the developing auditory system. This pathway provides a negative feedback control of IHC excitability by locally strengthening synaptic inhibition.

## Additional information

### Competing interests

None declared.

## References

[tjp12313-bib-0001] Chi DH & Kandler K (2012). Cannabinoid receptor expression at the MNTB‐LSO synapse in developing rats. Neurosci Lett 509, 96–100. 22230885 10.1016/j.neulet.2011.12.047 PMC3406926

[tjp12313-bib-0002] Kong JH , Zachary S , Rohmann KN & Fuchs PA (2013). Retrograde facilitation of efferent synapses on cochlear hair cells. J Assoc Res Otolaryngol 14, 17–27. 23183877 10.1007/s10162-012-0361-0 PMC3540278

[tjp12313-bib-0003] Kullmann DM & Lamsa K (2008). Roles of distinct glutamate receptors in induction of anti‐Hebbian long‐term potentiation. J Physiol 586, 1481–1486. 18187472 10.1113/jphysiol.2007.148064 PMC2375711

[tjp12313-bib-0004] Maiese K , Chong ZZ & Li F (2005). Driving cellular plasticity and survival through the signal transduction pathways of metabotropic glutamate receptors. Curr Neurovasc Res 2, 425–446. 16375723 10.2174/156720205774962692 PMC2258008

[tjp12313-bib-0005] Sendin G , Bourien J , Rassendren F , Puel JL & Nouvian R (2014). Spatiotemporal pattern of action potential firing in developing inner hair cells of the mouse cochlea. Proc Natal Acad Sci USA 111, 1999–2004. 10.1073/pnas.1319615111 PMC3918831 24429348

[tjp12313-bib-0006] Shen J , Harada N , Nakazawa H & Yamashita T (2005). Involvement of the nitric oxide‐cyclic GMP pathway and neuronal nitric oxide synthase in ATP‐induced Ca^2+^ signalling in cochlear inner hair cells. Eur J Neurosci 21, 2912–2922. 15978003 10.1111/j.1460-9568.2005.04135.x

[tjp12313-bib-0007] Steinert JR , Kopp‐Scheinpflug C , Baker C , Challiss RA , Mistry R , Haustein MD , Griffin SJ , Tong H , Graham BP & Forsythe ID (2008). Nitric oxide is a volume transmitter regulating postsynaptic excitability at a glutamatergic synapse. Neuron 60, 642–656. 19038221 10.1016/j.neuron.2008.08.025

[tjp12313-bib-0008] Ye Z , Goutman JD , Pyott SJ & Glowatzki E (2017). mGluR1 enhances efferent inhibition of inner hair cells in the developing rat cochlea. J physiol 595, 3483–3495. 10.1113/JP272604 PMC5451706 28211069

